# Animal Models of *Cryptococcus neoformans* in Identifying Immune Parameters Associated With Primary Infection and Reactivation of Latent Infection

**DOI:** 10.3389/fimmu.2020.581750

**Published:** 2020-09-15

**Authors:** Tyler G. Normile, Arielle M. Bryan, Maurizio Del Poeta

**Affiliations:** ^1^Department of Microbiology and Immunology, Stony Brook University, Stony Brook, NY, United States; ^2^Ingenious Targeting Laboratory Incorporated, Ronkonkoma, NY, United States; ^3^Division of Infectious Diseases, School of Medicine, Stony Brook University, Stony Brook, NY, United States; ^4^Veterans Administration Medical Center, Northport, NY, United States

**Keywords:** cryptococcosis, infection models, primary infection, granuloma, latent infection, immunodeficiency, host immune response

## Abstract

*Cryptococcus* species are environmental fungal pathogens and the causative agents of cryptococcosis. Infection occurs upon inhalation of infectious particles, which proliferate in the lung causing a primary infection. From this primary lung infection, fungal cells can eventually disseminate to other organs, particularly the brain, causing lethal meningoencephalitis. However, in most cases, the primary infection resolves with the formation of a lung granuloma. Upon severe immunodeficiency, dormant cryptococcal cells will start proliferating in the lung granuloma and eventually will disseminate to the brain. Many investigators have sought to study the protective host immune response to this pathogen in search of host parameters that keep the proliferation of cryptococcal cells under control. The majority of the work assimilates research carried out using the primary infection animal model, mainly because a reactivation model has been available only very recently. This review will focus on anti-cryptococcal immunity in both the primary and reactivation models. An understanding of the differences in host immunity between the primary and reactivation models will help to define the key host parameters that control the infections and are important for the research and development of new therapeutic and vaccine strategies against cryptococcosis.

## *Cryptococcus neoformans* is an opportunistic fungal pathogen

*Cryptococcus* spp. are basidiomycetes ubiquitously found within the environment as basidiospores and budding yeast, most commonly in the soil, trees, and avian habitations (1, 2). Of clinical relevance, two main species, *Cryptococcus neoformans* and *Cryptococcus gattii*, are encapsulated fungal pathogens and the etiological agents of cryptococcosis, a life-threatening invasive fungal disease that infects humans via the respiratory tract (3, 4). Immunocompetent hosts can be infected but rarely succumb to infection. Conversely, under conditions where the host cannot immunologically control the initial pulmonary infection, such as during antiretroviral therapy in HIV/AIDS patients, chemotherapy in cancer patients, or immunosuppressive therapy in organ transplant patients, the yeast may uncontrollably proliferate and disseminate to the central nervous system resulting in meningoencephalitis (5–7).

The *Cryptococcus neoformans/gattii* species complex has a high degree of heterogeneity, and has been classified by numerous molecular typing techniques (8, 9). These genotypic classifications have greatly aided in the epidemiology and genetic diversity that exists within these species, however the serotype classification of these species will be most relevant to this review since our focus is on the host immune response to the yeast. There are five serotypes of *Cryptococcus*, with serotypes A, D, and AD belonging to *C. neoformans* and serotypes B and C belonging to *C. gattii* (9–11). These serotypes are based on differences in the arrangement of the glucuronoxylomannan (GXM) capsule surrounding the yeast, which is considered a major cryptococcal virulence factor (12, 13). The various serotypes result in prominent differences in both pathology and immune modulation through variations in pattern-recognition receptor ligation and immune modulation with host cells. In fact, the GXM capsule was shown to be a major deciding factor in determining pathogenicity of the various species of *Cryptococcus* (14). For instance, the capsule of *Cryptococcus liquefaciens* (a non-pathogenic species) does not exhibit the same microbial defenses against amoebas as does the pathogenic species capsule. As such, these serotype differences play distinct roles in modeling host susceptibility and geographical distribution from the clinical side.

Due to its worldwide distribution, it is widely accepted that human subjects are exposed to this fungus during early childhood (15, 16), and upon this primary infection, the host harbors fungal cells in lung granulomas (17–30). Perhaps the best evidence that supports this possibility is provided by studies showing that fungal strains from patients affected by cryptococcal meningoencephalitis are identical to those strains isolated earlier from the same asymptomatic patients (31–33). Other investigators have suggested that these findings were the result of patients being constantly re-exposed to the very same strain from the environment (34, 35). Although this assertion is in the realm of possibility, it does not take into consideration the enormous genetic variability of cryptococcal strains present in the environment (36–38). Due to the evidence of strain diversity mentioned above, it is our opinion that the chance of inhaling a genetically identical strain years apart is less probable than the reactivation of a prior infection. In light of this, the evidence together strongly suggests that primary infection, granuloma formation, and eventual reactivation of the dormant yeast cells upon immunosuppression reflect the stages of this disease.

### Fungal Propagules: Spores vs. Yeast Cells as Infectious Particles

Pulmonary cryptococcosis begins upon inhalation of fungal particles, which can be either spores or/and yeast cells. The model organism, most commonly mice, receive these particles via intranasal or intra-tracheal challenge to recapitulate human infection (39). However, spores differ compared to yeast cells, and these differences may account for a different immune recognition and response to the infection (40).

Primarily, the spores of *C. neoformans* expose β-glucans, whereas encapsulated yeast cells expose the GXM capsule. β-glucans are strongly recognized by C-type lectin receptors (CLRs) on both resident and innate immune cells. The encapsulated yeast cells, however, weakly stimulate these same CLRs, as they ligate to TLR2, TLR4, CD14, and CD16 (41–44), resulting in different outcomes dependent on strain or host cell type.

Because of the pleiotropic effect of GXM and because GXM is a potent immunomodulator, yeast cells can induce a hyperinflammatory response that will eventually lead to the death of the host (particularly mice), or/and a non-protective type 2 immune response that will also be harmful to the host. Conversely, uptake of spores does not lead to a strong inflammation as spores can exist within macrophages for long periods of time without damaging them (13, 42, 45). Also, spores are less capable to stimulate adaptive immunity, which is well-known to be stimulated by GXM in encapsulated fungal cells through APCs, DCs, and macrophages (46–50). Thus, spores can evade host immunity much more efficiently than yeast cells, causing the development of a latent infection, and perhaps, they may represent a better model for studying the reactivation model. However, it is not known if spores are able to promote granuloma formation in mice and represents a topic for future investigation.

## Animal Models for Studying Cryptococcosis

There have been several approaches utilized to investigate human cryptococcal infection in the laboratory. Whole-organism approaches and fungal mutants (24, 46, 51–56), *in vivo* antibody depletions and neutralizations (20, 56–61), and the assessment of fungal burden, host survival, and immune cell recruitment (present in nearly all *C. neoformans* studies) have been at the forefront of these modeling approaches with each uncovering notable findings as well as bringing limitations.

### Invertebrate Models

Both invertebrate and vertebrate models exist in the literature for *in vivo* modeling of *C. neoformans* infection. This topic has been reviewed in the past (62), and this review will expand on the ample research that has been conducted since then. Invertebrate models include nematodes (*Caenorhabditis elegans*), amoebae (*Acanthamoeba castellanii* and *Dictyostelium discoideum*), and insects (*Drosophila melanogaster* and *Galleria mellonella*). The relatively low cost of maintenance, lesser ethical restrictions, ease of genetic manipulation, and short reproduction times makes invertebrates valuable tools in biomedical research. Research with invertebrate models has mainly addressed mapping signal transduction pathways for virulence during infection and the interactions of *C. neoformans* with innate phagocytic cells since invertebrates possess only the innate arm of the immune system (63). In light of invertebrate models, several studies have highlighted these organisms as practical tools.

First, Mylonakis et al. found that *C. elegans* can use non-pathogenic species of *Cryptococcus* (*C. laurentii* and *C. kuetzingii*) as food sources while *C. neoformans* resulted in death of the worms (64). In addition, the *C. neoformans* gene LAC1 was a virulence factor upregulated during infection of the worms, which confirmed prior mammalian studies as well as added to the growing belief that *C. neoformans* may have evolved into a pathogen during interactions with environmental predators such as *C. elegans*. From another group, *C. elegans* was used as part of a multi-host screen of *C. neoformans* mutants to assess previously unknown virulence factors (65). Several genes regulating lipid metabolism, chitin regulation, and melanin synthesis were discovered using this approach. These studies among others support *C. elegans* as a viable model for assessing *C. neoformans* infection in the lab.

Second, soil amoeba are reservoirs of *C. neoformans*, and resemble the way macrophages phagocytose microorganisms making them an important tool in *C. neoformans* research (17, 66, 67). A 2018 paper showed that *D. discoideum* phagocytosed *C. neoformans* but was unable to kill the yeast, a phenomenon similar to macrophages. However, Watkins et al. were able to ascertain that *C. neoformans* can either be expelled from the amoeba, or when the amoeba is pharmacologically blocked, *C. neoformans* can escape in a non-lytic manner (68), a recent phenomenon termed vomocytosis (69, 70). Ultimately, the amoeba model represents a novel approach for future studies on the cellular level, which has implications to how macrophages interact with *C. neoformans* in the host.

Finally, insects represent a valuable experimental tool for studying how innate phagocytes interact with the yeast cells as well as the effect of antimicrobial peptides on yeast viability (63, 71). *G. mellonella* can live at human body temperature and be infected with controlled doses of the yeast with minimal invasion. This is in contrast to the models above since *C. elegans* are inoculated onto an agar plate with colonies of *C. neoformans* and there is no way to regulate the infection dose and timing. This organism has been used primarily to assess virulence of *C. neoformans* (46, 65) and antifungal susceptibility (72). Altogether, *G. mellonella* represent one of the most versatile invertebrate tools for *C. neoformans* infection studies.

### Vertebrate Models

Despite the progress that has been made utilizing invertebrate organisms, there are several limitations to their use as a model for cryptococcosis. One major limitation is the lack of an adaptive immune system, which has been repeatedly shown to be instrumental in host protection against *C. neoformans*. Secondly, the route of infection for invertebrates does not recapitulate the inhalation infection model in humans. Finally, a major immunological limitation of invertebrate organisms is that they lack differentiated phagocytes, such as macrophages and DCs and only have a generalized phagocyte cell type (73). However, these limitations can be overcome using vertebrate organisms as model hosts that offer a vast selection of tools. Primarily, the presence of an adaptive immune system and differentiated cell types, easy regulation of infection doses, and availability to infect via inhalation all offset the aforementioned invertebrate disadvantages. Additionally, the larger body sizes of these animals allow for more experimental manipulation to be carried out, such as endotracheal intubations, radiography and imaging, bronchoalveolar lavage, and cerebrospinal fluid collection (63), and the presence of organ systems allows for an infection model that closely resembles how humans become infected and succumb to CNS dissemination. As with all model hosts, each brings advantages as well as limitations, all of which will be briefly discussed below.

Several vertebrate organisms have been used in the modeling of *C. neoformans* infection, and some of these include zebrafish (*Danio rerio*), non-human primates, rabbits (*Oryctolagus cuniculus*), rats (*Rattus rattus*), and most commonly mice (*Mus musculus*). The zebrafish is a relatively newer host model for *C. neoformans* infection, although it has been established in other host-pathogen interactions and offers an exciting middle ground between the simplicity of invertebrate models and the organ system complexity found in mammals (74). Obvious advantages of this host include the optical transparency for live imaging where the yeast have been observed replicating in macrophages, genetic amenability for host mutational studies, and assessment of virulence for different strains of *C. neoformans* (75–77). However, certain elements must be considered when using this model. First, the authors reported that neutrophils did not accumulate at the site of infection or around infected macrophages in the zebrafish model (77). Although the role of neutrophils in mammalian infections is still uncertain, the early accumulation of neutrophils is a well-established immune signature for *C. neoformans* infections in mice and humans. Secondly, only three studies using zebrafish to model *C. neoformans* infection (and none using *C. gattii*) having been published to date, so more data are necessary for a thorough understanding of zebrafish-*Cryptococcus* interactions.

Non-human primates are an uncommon host for studying cryptococcosis. This is most likely due to the greater cost and housing requirements compared to other animal models (78, 79). Most studies in non-human primates were conducted decades ago, but a recent 2019 study performed a transcriptome analysis of cynomolgus monkeys (*Macaca fascicularis*) and mice using RNA-Seq during acute *C. neoformans* infections (80). The authors found that only about 20 percent of the differentially expressed genes were shared between these two hosts during infection, and they suggested monkeys could be a better model than mice at recapitulating the human response to *C. neoformans*. Although additional host data adds to the growing body of cryptococcal literature, this small sample size of monkeys (6 total monkeys; 3 control and 3 experimental) in one study does not justify the expense and challenge of using monkeys as hosts to *C. neoformans*.

Historically, rabbits have not been a commonly used host for studying cryptococcosis mainly because of the high cost to purchase and maintain, however the rabbit model closely mimics the human infection. Rabbits are naturally resistant to *C. neoformans* and they succumb to the fungus only when immunosuppressed (e.g., with corticosteroids) (81). The rabbit model is suitable for the introduction of fungal cells directly into and out of the subarachnoid space, allowing studies that address the fitness and the adaptation of fungal cells to the cerebrospinal fluid. This is a particular strength of this model because the most common clinical manifestation of cryptococcosis is meningitis. Understanding how fungal cellular pathways respond to this unique environment may provide important insights into the development of new therapeutic strategies targeting those specific pathways. Rabbits are also used to test the efficacy of new antifungals at the site of infection during cryptococcal meningitis (82–85). Thus, rabbits are a tractable alternative for studying cryptococcal meningitis.

Rats have been reported to develop chronic pulmonary cryptococcosis in the wild (86). Rat models of cryptococcal virulence and infection were more prevalent in the late 1980's, 1990's, and early 2000's (87–94), but have diminished over the years. *In vivo* and *ex vivo* studies using rats have mainly focused on *C. neoformans* interactions with alveolar macrophages (95–97). Similar to the rabbit model, rats also have the ability to form lung granulomas that efficiently contain *C. neoformans* within the granuloma until the animal becomes immunocompromised, which recapitulates the human pathophysiology [(87, 88, 92, 94)]. Rats are also a valuable model for *C. gattii* infections since they are naturally susceptible hosts and *C. gattii* infections are more common in immunocompetent humans (98–100). Although rats represent a well-defined host to cryptococcal infections, the limitations for this model are the cost and the lack of genetic knockout animals for studying the role of host parameters against cryptococcosis.

Notably, the mouse represents by far the most well-documented animal model to study host-pathogen interactions with *C. neoformans*. The mouse offers great flexibility for experimental studies with a large diversity in genetic knockout tools commercially available. In addition, the mouse has been well-characterized in biomedical research and ease of handling with numerous routes of infection. Nevertheless, discordance remains when distinguishing between the primary infection model and reactivation model mostly due to lack of tools used to study the reactivation model of infection in mice.

Differently from humans, rabbits, and rats (92, 94, 101–105), mice do not produce a granulomatous response against highly virulent *C. neoformans* strains, and they eventually succumb to the infection. Investigators have utilized less virulent strains, such as *C. neoformans* strain 52D, in which mice develop a persistent infection with a granulomatous response after intranasal infection (106–110). This model has allowed researchers to study chronic cryptococcosis and brain dissemination in mice, although there are several drawbacks to using less virulent strains that include induction of immunity not normally associated with highly virulent strains. Thus, due to a model that does not fully recapitulate human granuloma containment of the yeast, nearly all work has focused on the primary infection model of cryptococcosis.

However, our lab has developed a mouse model of cryptococcosis that recapitulates the human response to *C. neoformans*. In fact, we found that mice infected with an avirulent strain lacking the sphingolipid glucosylceramide (Δ*gcs1*) leads to total containment of fungal cells in lung granulomas and no dissemination to the brain (111). However, when Tgε26 mice who are inherently immunocompromised (Tgε26 mice lack T and NK cells) are instranasally infected with this mutant, the mice do not form lung granulomas, the Δ*gcs1* mutant uncontrollably proliferates in the lung, disseminates to the brain, and results in complete death of these mice (20). Thus, this mutant mimics the physiopathology of the infection in humans with which cryptococcosis is mostly associated (112).

Although many animal models are available, the majority of immunological studies have been performed in mice, so the following sections of this review will focus on discussing key immunological findings in mice for both the primary infection model and reactivation model of cryptococcosis.

## Modeling Cryptococcal Infections and What Has Been Learned

**Figure 1:**
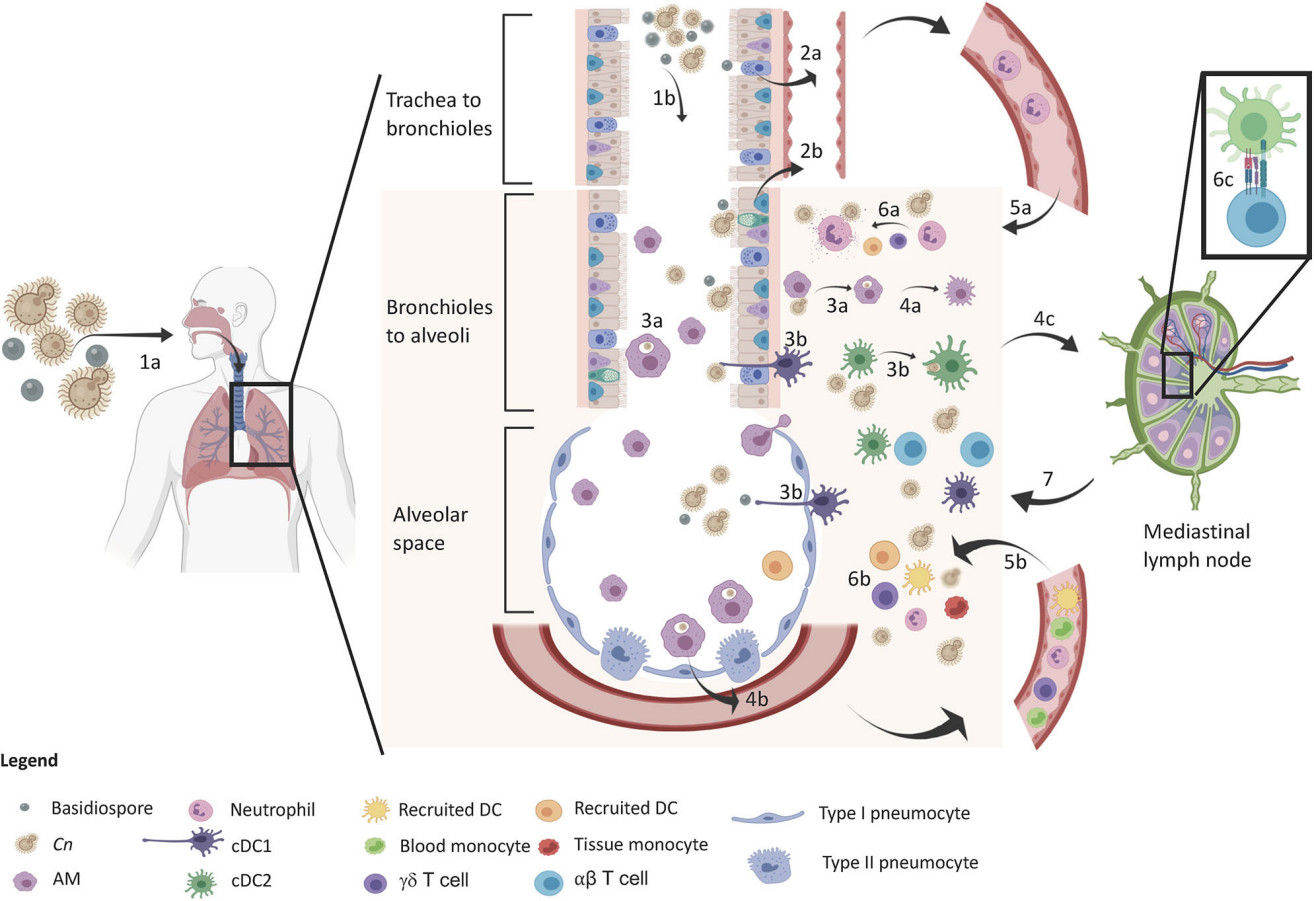
Protective immune response in the lung upon inhalation of *C. neoformans*. The sequence of events is denoted in the diagram. The yeast and basidiospores are inhaled (1a) and travel down the airways passing through the trachea to the bronchioles (1b) and further down to the alveolar spaces. Upper respiratory epithelia sense the spores (2a) and lower respiratory epithelium sense spores and encapsulated propagules (2b), which release IL-8 for early neutrophil recruitment. Macrophages (3a) and DCs (3b) phagocytose *Cn* in both the airways and lung tissue leading to M1 macrophage polarization (4a) and cytokine release (GM-CSF and MCP-1) for inflammatory cell recruitment (4b). DCs mature and migrate to the lung draining lymph node (4c) for induction of adaptive immunity. At this time, neutrophil (5a) and inflammatory cells (5b) infiltrate into the lung tissue for increased host defense against *Cn*. Neutrophils kill *Cn* via degranulation (6a) and a myriad of other defenses from other cells (6b) such as monocyte differentiation into recruited DCs that further amplify T cell induction (6c). Type 1/17 polarized T cells migrate back to the lung for adaptive immune control of infection leading to control/containment of the infection.

In the primary infection model to cryptococcosis, the host is exposed to the fungus for the first time upon inhalation of spores and/or desiccated yeast cells. It has been postulated that the primary infection happens very early on in life since there are data showing children having antibodies to the yeast in their blood [(16, 113)]. Protection against cryptococcosis is dependent on early host recognition, recruitment of proper cell types, and immunological control of yeast proliferation (114–116). The immune response to *C. neoformans* therefore can be temporally divided into three stages: (1) recognition of the inhaled particles by resident airway cells, (2) early recruitment of innate immunity, and (3) late recruitment of adaptive cell-mediated immunity (Figure 1). These stages collaboratively work in an orchestrated manner to control the pulmonary infection and deter extrapulmonary dissemination and are discussed in the following sections.

**Figure 2:**
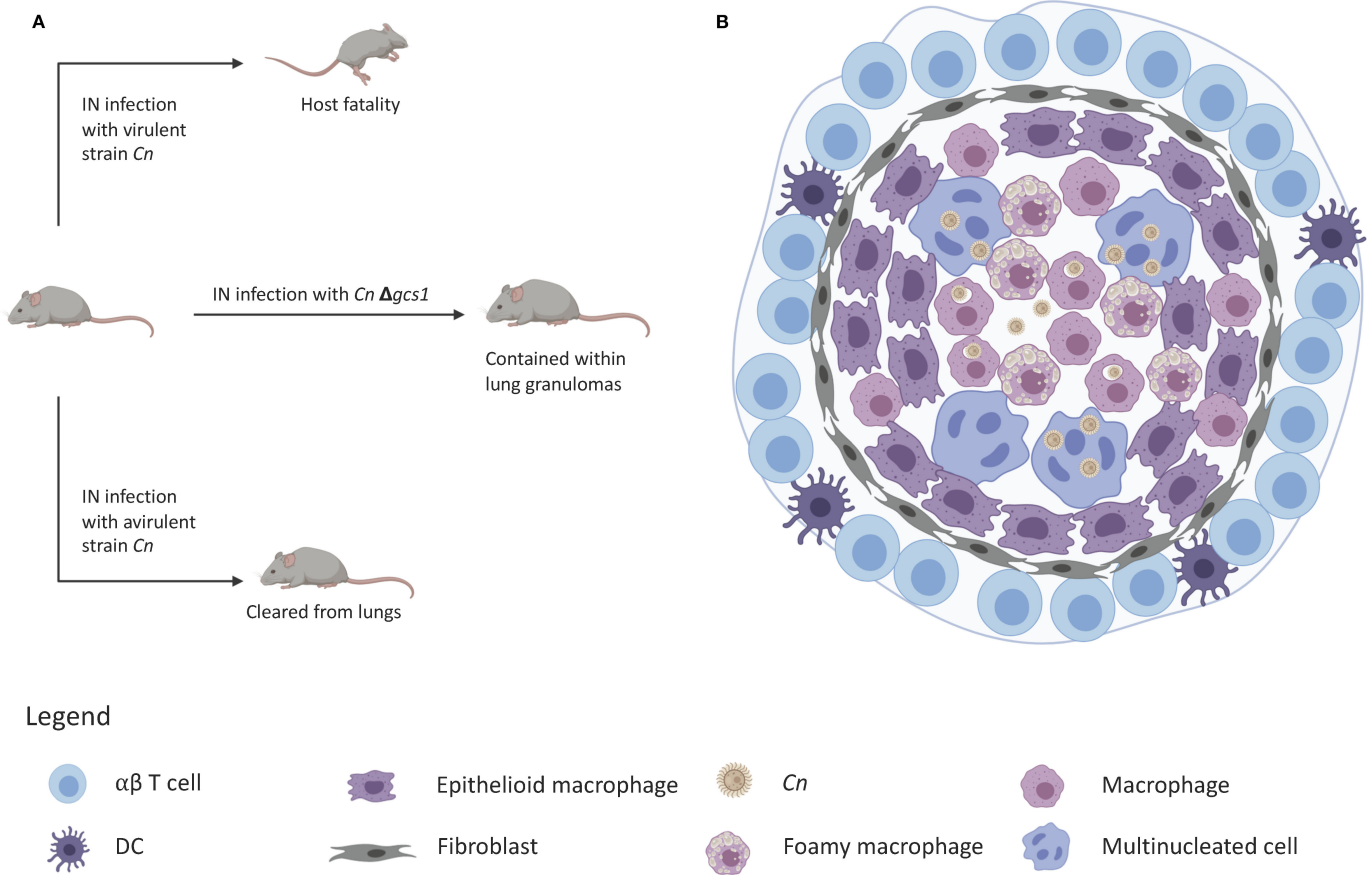
Granuloma containment in mice infected with *C. neoformans*△*gcs1*. **(A)** Mice are notorious for lacking the ability to form lung granulomas to contain *C. neoformans* infection. Infection with a virulent strain results in death and infection with an avirulent strain leads to clearance of the yeast from the lungs. Infection with *C. neoformans*△*gcs1* results in granuloma containment of the yeast in the mouse lungs. **(B)** These granulomas exhibit a slightly necrotic center with macrophages, foamy cells, and giant multinucleated cells with internalized *C. neoformans*. These cells are surrounded by a closure of epithelioid cells and fibroblasts, followed by an outer ring of lymphocytes.

As previously mentioned, upon initial inhalation of *C. neoformans*, immunocompetent hosts generally control the infection, but the fungus is not always fully cleared from the lungs. In humans, *C. neoformans* can remain in a latent state of infection contained within lung granulomas, which are localized structures composed of several cell types that work in an coordinated manner to contain the pathogen (Figure 2B) (117). Using two strains of rats, it was shown that a type 1 immune response was needed to control pathogen replication and granuloma containment of *C. neoformans*, and that a type 2 immunity resulted in loss of fungal containment with the consequent exacerbation of the disease (88, 92). Although the host does not experience any pathology during latency, granuloma containment introduces a paradoxical situation: on the one hand these structures help to contain the infection, but on the other hand they also harbor the pathogen and provide a source of fungal replication once immunodeficiency occurs, such as upon HIV/AIDS-mediated lymphopenia (118, 119) or solid organ transplant recipients (4, 117). The immunological events associated with the reactivation model will be discussed following the primary model as well as current progress in a novel mouse model of cryptococcal granuloma containment.

### Primary Infection Model: Inhalation of the Fungal Particles

#### Airway Resident Cell Recognition of *C. neoformans*

In the absence of infection, an organism still has immune surveillance for early detection and response to foreign particles. This is especially exhibited at mucosal sites such as the intestines, skin, and the airways. Airway resident immune cells include the epithelial cells that line the airways, tissue-resident alveolar macrophages and dendritic cells, and non-canonical innate lymphocytes such as γδ T cells and innate lymphoid cells.

##### Epithelial cells

The inhalation of the fungal propagules, the first host cells to encounter these particles would naturally be the airway epithelium and resident airway immune cells. Airway epithelial cells serve as a barrier and immunological interface between the external and internal bodily environments (120–123). In depth studies on cryptococcal-epithelial interactions are lacking in relation to other cell types, but both the upper respiratory bronchial epithelia and lower alveolar epithelia have been shown to recognize, respond to, and internalize *C. neoformans* (124–127). Utilizing *in vitro* approaches, the human type II alveolar epithelial cell line, A549, several groups came to find that both GXM (128) and phospholipase B (124) were epithelial adherence factors. In fact, the adherence to lung epithelial cells (using A549 cell line) can be blocked by either using an antibody against the cryptococcal capsule (anti-GXM antibody, 18B7), or by using a Δ*plb1* mutant (lacking fungal phospholipase b). The cryptococcal capsule is clearly important for the adherence to A549 but its role is less clear because acapsular mutant (Δ*cap67*) is still able to attach to A549 and this attachment was only inhibited in the presence of specific fungal mannoprotein (s) (e.g., MP84) (126). Upon adherence, internalization of *C. neoformans* by the airway epithelial will follow, and although the fungal and host factors regulating cryptococcal-epithelial interaction is still under investigation, one thing is clear: internalization of cryptococcal cells may cause death of the epithelial cells resulting in lung lesions and hyperinflammation (122).

Upon adherence, lung epithelial cells actively participate in the immunological response via secretion of specific cytokines. For instance, *in vitro* studies showed that when GXM binds to CD14 on A549 cells, they secrete IL-8 (41). IL-8 is a potent neutrophil recruitment chemokine, suggesting that lung epithelium may drive early recruitment of these early host effector cells. This paper was shortly followed by another study that used the BEAS-2B cell line that more closely mimics the upper bronchial epithelium, an area that would obviously be exposed to inhaled propagules first (127). This group also found that IL-8 was secreted in an NF-κB dependent manner in response to *C. neoformans* infection, but BEAS-2B cells only responded to an acapsular mutant and not the capsulated parent strain. It is possible that the different responses observed was due to the different cell types used (A549 vs. BEAS-2B), but another possibility is that upper bronchial epithelial cells respond more effectively to acapsular (or a thinner capsule) cryptococcal cells compared to epithelial cells found in the lower alveolar epithelium. It has been shown that cryptococcal particles have a very small or no capsule upon inhalation, and rapidly induce capsular growth as they proceed down the airway (42, 129–131). One final possibility stems from the limitations and drawbacks some researchers find in using the A549 cell line. Phenotypic studies of A549 cells showed they possess lower phospholipid content, fewer cytoplasmic lamellar bodies, and a higher autophogy rate compared to primary human type II alveolar cells (132, 133). Nonetheless, this evidence together with the cytokine data clearly suggests that the upper respiratory tract may sense less capsulated particles and respond by producing cytokines which recruit neutrophils early on, while differential responses may occur after capsule synthesis has been stimulated further down the airway.

Capsule synthesis consists in the production of a well-organized physical barrier containing glucuronic acid, xylose, and mannose (GXM) molecules connected through various glycosidic bonds. GXM strongly inhibits the protective host response to cryptococcal infection via initiation of a type 2 immunity (13, 42). Several groups have described the release of IL-33 in response to epithelial-cryptococcal interactions and the downstream effects of IL-33 through its receptor T1/ST2 (134, 135). IL-33 drives a type 2 immunity via the activation of type 2 innate lymphoid cells and promoting a Th2 T cell response in the lungs. These effects lead to an overabundance of IL-5 and IL-13 in the lung resulting in alternative M2 macrophage polarization and decreased epithelial barrier integrity promoting fungal cell persistence and penetration into the lung tissue and lung blood vessels. These negative facets may eventually lead to the dissemination of fungal cells from the lung to other organs through the bloodstream including the CNS. Interestingly, the source of IL-33 in the lungs was found to be from type II alveolar cells (135), the same source of IL-8 mentioned above. Specifically, IL-33 downregulates surface receptor expression for leukocyte extravasion, such as E-cadherin and ICAM-1, which decreases the recruited immune cell response. The protective (IL-8) and non-protective (IL-33) results together demonstrate the varied response to inhaled cryptococcal particles suggesting a strong dependence on strain, capsule size, and experimental conditions for host outcome.

##### Alveolar macrophages

In addition to the airway epithelial cells, CD11c^+^ airway resident immune cells such as alveolar macrophages (AM) and DCs have been reported to be the dominant cell populations in uninfected lungs (47, 136–139) and have been arguably the most widely studied cell population in the cryptococcal literature (Figure 1). These cells are found ubiquitously in the lower respiratory and alveolar spaces of the airway lumen and the underlying lamina propria below the epithelium. These pulmonary sentinels phagocytose debris, dead cell material, and inhaled particles found in the airways for removal as well as add to the early cytokine response in the lungs during the early phases of infection such as with *C. neoformans*.

AM have been suggested to play paradoxical roles during infection with *C. neoformans* (140, 141), so that the necessity of these cells to efficiently control *C. neoformans* lung infection has been strongly debated (142). On one hand, depletion of these cells (using anti-CD11c) prior to infection exhibited both increased lung fungal burden and the mortality rate in mouse models (50). On the other hand, *C. neoformans* is clearly capable of living within AMs (and other macrophages populations) unharmed shown by numerous studies (69, 143–146). It is important to note, however, that AMs are tissue resident macrophages in the lungs, but during an infection, a collection of macrophages consisting of AMs, interstitial macrophages, and monocyte-derived macrophages will populate the lungs. This collection, referred to in this review as pulmonary macrophages, needs to be set apart from studies looking at AMs alone.

Pulmonary macrophages have been shown to shift from an unpolarized state in uninfected animals to either classically activated M1 or alternatively activated M2 phenotypes depending on the lung cytokine microenvironment during infection (147–151). These polarization states dictate the effector functions of these cells. M1 macrophages are activated by IFN-γ and have been widely regarded as anti-cryptococcal in nature, while M2 macrophages become activated by IL-4 and are regarded as non-protective during infection with *C. neoformans*. M1 macrophages are regulated by STAT1 and are an early source of inflammatory cytokines and chemokines, such as IFN-γ, TNFα, IL-1, and IL-8, that drive the protective type 1 immune response against cryptococcosis (53, 137, 152–154). More specifically, M1 macrophages exhibit increased phagocytosis (147, 153), killing of *C. neoformans* via ROS/NOS production (53, 153), and aid the maturation of dendritic cells (DC) to activate adaptive cell-mediated immunity. Interestingly, these macrophages have also been reported as having innate memory in immunization studies (53). Through IFN-γ-primed macrophages from the IFN-γ-producing strain used in the Wormley lab, the authors observed an increased STAT1 binding to the promoter region and increased expression of antifungal gene expression. Thus, M1 polarized macrophages possess the ability to control *C. neoformans*, coordinate a protective response in the host, and may possess memory to future infections.

In contrast to anti-cryptococcal M1 macrophages, M2 polarized macrophages are regulated by STAT3, produce IL-5 and IL-13, and drive a type 2 immune response in the host, which is unable to contain the infection (147, 155). Because *C. neoformans* can survive and replicate within macrophages (13, 156, 157), M2 macrophages that have little to no fungicidal activity can harbor large numbers of yeast cells leading to dysfunctional macrophages (69, 145). Through either lytic or non-lytic (termed vomocytosis) exocytosis, cryptococcal cells may exit the macrophages either in the lungs or elsewhere if the macrophages have also left the lungs (70, 146, 158). This ability of *C. neoformans* to “hide” within macrophages protects the yeast against the harsh extracellular environment containing antimicrobial peptides and complement as well as killing by neutrophils, NK cells, and T cells (75). Additionally, the harsh environment of the phagosome may lead to the formation of titanized cryptococcal cells with consequential macrophage disruption and reduction in phagocytosis by other macrophages or/and extracellular killing of these titan cells (12, 159–162). Eventually, this leads to the persistence of the infection instead of containment or clearance.

These differential findings with AM in mice have also been observed with rat and human macrophages (94, 95, 97, 163–167). Early studies showed that *C. neoformans* was rapidly ingested by human AM, although no killing was observed, and the yeast were able to proliferate over the course of 6 h or 2 days (164, 166). The 1994 study also showed that human AM played a key role in antigen presentation to T cells. In rats, three studies from the same group in 1989 identified that rat AM phagocytose and effectively kill *C. neoformans* in the presence and absence of serum using *in vitro* assays (95–97). Taken together, the role of AM seems to be a variable response with respect to *C. neoformans*, especially when comparing *in vitro* and *in vivo* assays and between different model organisms.

Despite a large body of work studying alveolar macrophages in cryptococcosis models, several questions remain for future research in what determines M1 vs. M2 macrophage polarization. Indeed, serotype differences in the GXM capsule and certain cytokine profiles in the lung have been suggested to play a role in driving macrophage polarization (43, 44, 168, 169). Research on how other fungal factors and the lung microbiome influence the fate of AMs (and more generally pulmonary macrophages) during primary infection and reactivation is still greatly needed.

##### Resident dendritic cells

Lung resident DCs express CD11c and MHC-II markers and have been highly implicated in early defense against invading pathogens and have gained an increased appreciation in antifungal immunology over the last decade. DCs are innate phagocytes with a central role to bridge the innate and adaptive arms of the immune systems upon initial infection with a pathogen, including *C. neoformans* (47, 170). Phagocytosis of the yeast requires complement or antibody-mediated opsonization of the GXM capsule (116, 171), and the DCs kill *C. neoformans* through lysosomal degradation (47). Concurrently, DCs will upregulate co-stimulatory molecules such as CD80 and CD86 for antigen presentation to T cells. DCs are a diverse cell type with several subsets each possessing specialized functions and regarded as essential components of antifungal immunity including against *C. neoformans*. Three main subsets can be found within the lungs: CD103+ classic DCs (cDC1), CD11b+ cDC (cDC2), and plasmacytoid DCs (pDC) (47, 116, 172).

cDC1 cells are under the regulation of the Batf3 transcription factor (173) and are predominantly found within the airways and alveoli of the lungs (Figure 1). These cells sit at the epithelial interface and directly interact with the external microbial cells present in the airway lumen. These cells have been described to help drive a type 1 immune response via the release of IL-12 and stimulation of NK cells and ILC1s, recognize intracellular pathogens, and cross-present antigens to CD8+ T cells (173). cDC1 become activated in response to pathogen uptake and degradation and cytokine exposure (such as TNFα and GM-CSF), upregulate costimulatory molecules (CD40, CD80/86, MHC-II) and migratory receptors (CCR7). They can travel to the lung-draining lymph node via a CCL21 gradient to potentiate protective T cell-mediated immunity to cryptococcal infection (61, 174, 175).

cDC2 cells are under the regulation of the IRF4 transcription factor (176) and are also found within the lower respiratory tract and alveoli but deeper in the lamina propria tissue below the basement membrane of the epithelial cells (177). These cells have been shown to play a role for induction of both Th2 and Th17 CD4+ T cell-mediated immune responses depending on infectious antigen type and cytokine environment (174, 176) in a similar manner to cDC1 cells described above. While the Th2 induction mechanism by these cells is still unknown, the Th17 response is mediated through release of IL-23, IL-6, and TGF-β (all the cytokines needed for type 17 immune activation and sustainability). Additionally, cDC2 aid in inflammatory cell recruitment via secretion of IL-12, MCP-1/CCL2, MIP-1α/CCL3, MIP-1β/CCL4, and RANTES/CCL5 (172, 178, 179), and this subset has also been described to be essential for iBALT (inducible bronchus associated lymphoid tissue) maintenance with the release of CXCL12, CXCL13, and CXCL15 being the primary factors (178, 180). Overall, this subset exhibits highly diverse functions in the lung that help drive anti-cryptococcal immunity and containment.

Finally, pDCs have been studied far less in comparison to their classic DC counterparts and represent only a small percentage of the DCs in the lung. The main area of research for this cell type is in viral infections since they are the strongest producers of type I interferons (177, 181) but are also implicated in response against bacterial infections. However, pDC-related work in fungal immunity is relatively low with only one study for pDCs response to *C. neoformans* has been published to date. This study by Hole et al. (182) showed that pDCs exhibit direct killing ability toward *C. neoformans* via ROS production and this killing was dependent on the dectin-3 receptor. However, dectin-3 KO mice were not hypersusceptible to *C. neoformans* infection compared to WT mice, suggesting that although pDCs can kill the yeast, their killing ability though dectin-3 receptor is not essential for survival.

##### γδ T cells

γδ T cells and innate lymphoid cells (ILCs) have been often overlooked in anti-cryptococcal defense, although both cell types have been described to be critical players in mucosal immunity to other pathogens and diseases. γδ T cells express a non-canonical TCR bearing γ and δ subunits and can be found as both blood circulating and tissue-resident subsets. These cells are strongly defined by their ability to secrete IL-17 upon infection (183–185).

For the work associated with *C. neoformans* infection, there are two papers that report differing conclusions. In the first paper from 2004, Uezu et al. infected WT and TCRδ^−/−^ KO mice (C57 background) with *C. neoformans* and assessed fungal burden and cytokine production between the two groups (186). The TCRδ^−/−^ knockout mice cleared the infection quicker than WT mice and showed higher IFN-γ production by cells in the draining lymph nodes. These results suggest that γδ T cells hinder host protection to *C. neoformans* infection in this mouse model.

The second paper published 8 years later looked at the role of γδ T cells in mice (Balb/c) depleted of neutrophils (60). The authors found that γδ T cells were a significant source of IL-17A in the lungs during neutropenia. It was concluded that γδ T cells were a viable source of IL-17A in the absence of neutrophils and may be a significant source of leukocyte recruitment early on during infection with *C. neoformans*.

The differences between the role of γδ T cells in these studies may be attributed to the different cryptococcal strains, mouse genetic backgrounds, or experimental approaches. Although both groups used serotype A strains of *C. neoformans*, Uezu et al. used a clinical isolate in C57 mice while Wozniak et al. used an IFN-γ producing mutant derived from the H99 strain in Balb/c mice. C57 mice are genetically more susceptible to *C. neoformans* infection since they rapidly induce eosinophilia, whereas BALB/c mice are more resistant to *C. neoformans* (39). In addition, the H99-γ mutant induces strong immunity in mice, even protecting them against subsequent lethal challenges. Thus, the use of this protective strain may have skewed the host into a more beneficial outcome compared to a more virulent strain. Despite this caveat, the findings contribute key knowledge as IL-17 is a widely accepted anti-fungal immune response and γδ T cells respond early to pulmonary infection suggesting these cells could play an instrumental role early on during infection with *C. neoformans*.

Since reactivation of *C. neoformans* is a common occurrence associated with HIV/AIDS patients, it is worth noting that HIV progression downregulates the number of circulatory γδ T cells (187). Moreover, the antiretroviral therapy (ART) does not reconstitute these γδ T cells. Thus, stimulation or/and introduction of γδ T cells could be an effective immunotherapeutic approach to combat HIV and cryptococcosis.

##### Innate lymphoid cells

ILCs are one of the most recently emergent cell populations in immunology research. This widely heterogeneous group of cells reside at barrier regions, such as the skin and mucosal surfaces of the lung and intestines, and perform a wide variety of tasks including tissue remodeling, lymphoid tissue biogenesis, and early immunological responses to infection (183, 188–190). There are three major groupings of ILCs that are found in the lung parenchyma. Their nomenclature represents the type of immunity they provide and will be briefly mentioned for clarity. Type 1 ILCs that include natural killer (NK) cells and ILC1, which are defined by their expression of Tbet and secretion of IFN-γ in response to IL-12. Type 2 ILCs that are defined by expression of GATA3 and secrete IL-4, IL-5, IL-9, IL-13, and amphiregulin in response to IL-25 and IL-33. Type 3 ILCs that include lymphoid tissue inducer (LTi) cells and both NCR+ (CD335; NKp46) and NCR- subsets of ILC3, which are defined by expression of RORγt and secretion of IL-22 and IL-17 (as well as small amounts of IFN-γ) in response to IL-1β, IL-23, and IL-6 (188, 189, 191). Together, ILCs represent a group of cells that respond very quickly upon infection with great variety, which is dependent on the subset that becomes stimulated.

Innate lymphoid cell studies with regards to *C. neoformans* have been largely lacking until two recent studies have highlighted an important role for ILCs in the host response to *C. neoformans*. Prior to 2018, it was known that type 1 ILC NK cells were able to recognize, become activated in the presence of, and directly kill *C. neoformans* due to the activating receptor NKp30 in both mice (192, 193) and humans (194) but the fungal ligand responsible for this cytotoxicity was unknown. Additionally, NK cells were found to be defective in HIV-patients. Over two decades later, Li et al. came to find that the ligand to NKp30 was β-1,3-glucan using a variety of approaches with *C. neoformans* and *Candida albicans* (195). They found that type 1 ILC NK cells bind directly to β-1,3-glucan, increasing expression of perforin upon binding, and that the exogenous addition of β-1,3-glucan restored the anti-cryptococcal killing ability of NK cells in HIV-infected patients. This study provided exceptional information for an understudied ILC cell type that added to the mouse and human models of immunological protection.

Very recently, Kindermann et al. looked into the early response defining the protective type 1 immune protection against *C. neoformans* (196). Using RORα knockout mice (deficient in ILC2s), the group saw a downregulation of type 2 cytokines, IL-4, IL-5, and IL-13, decreased number of eosinophils, and significantly lower fungal burden compared to the WT C57 mouse control. This was accompanied by an increase in classical M1 macrophage activation, ultimately showing the host responded with a protective immune response. Lung histopathological analysis confirmed that there was reduced lung tissue damage associated with the increased type 1 immunity. These results further confirm the hypothesis that an ILC2 response may lead to a detrimental host outcome during cryptococcal infection (135). This study confirmed prior findings suggesting IL-33 preferentially increased ILC2 proliferation in the lung and downstream IL-13 release. Overall, these results suggest that ILC2s preferentially activate in response to highly virulent strains of *C. neoformans* during early infection timepoints to stimulate a non-protective, type 2 immune response.

#### Recruited Innate Immunity to *C. neoformans*

Inflammatory cell recruitment is essential for early control of *C. neoformans* infection (114, 197, 198). Neutrophils and dendritic cells have both been extensively studied and shown to play positive roles in the early host defense, while monocytes and eosinophils have been reported to play controversial or negative roles during infection with *C. neoformans*.

##### Neutrophils

Neutrophils are granulocytes originating in the bone marrow that are among the first inflammatory cells to respond to infection in great numbers and take part in a type 17 immune response (199–202). These cells exhibit a short lifespan and offer a myriad of defense mechanisms. Neutrophils have been widely implicated in anti-fungal defenses, especially against *Candida albicans* and *Aspergillus fumigatus* (136, 199, 203, 204), but since neutropenia is not a risk factor to developing cryptococcosis, the role of neutrophils has been sparsely explored in response to *C. neoformans*. However, the question remains if neutrophils are an important protective cell type even if the loss of them does not render host susceptibility.

Neutrophils have been suggested to play an important role for protection in humans once the infection has occurred. This defense stems from observations from several reports showing (i) dampened killing ability of macrophages and neutrophils during late stages of lymphopenia such as with AIDS progression (205, 206); (ii) impaired activation and effector functions of human neutrophils in response to TNF-α, IL-1β, and nitric oxide deficiency was observed in healthy patients who succumbed to pulmonary cryptococcosis (207); (iii) although neutropenia is not a risk factor for cryptococcosis, neutropenia is commonly observed in patients with HIV/AIDS (206, 208, 209). Taken together, these early studies strongly implicate human neutrophils as playing a role in anti-cryptococcal defense.

These data do not fully hold true in mice since murine neutrophils are known to be weaker than human neutrophils since they lack fully activated defensins (210). Neutrophils have been shown to exert a protective role during pulmonary infection caused by several microorganisms (211–213), but the role of neutrophils during *C. neoformans* infection remains unsettled in mice.

Neutrophils internalize *C. neoformans*, respond with the release of effector cytokines most notably IL-17A, kill the yeast cells via oxidative bursts and toxic cytoplasmic degranulation (214). Only a very limited amount of published work has addressed the role of neutrophils in animal models and depending on the animal model, strain of *C. neoformans*, and/or route of infection, the results seem to differ (50, 60, 215, 216). For example, mouse strains more resistant to *C. neoformans* (SJL/J) seem to benefit from the presence of neutrophils (216) but more susceptible strains (BALB/c) exhibited enhanced resistance upon neutrophil depletion (215). These studies were both conducted using the weaker *C. neoformans* strain D52. In addition, the BALB/c mice were depleted of neutrophils prior to infection but not continued throughout the course of infection. The number of neutrophils matched the control mice by day 7 post infection, so the role of neutrophils in the overall host outcome is not able to be formulated. Thus, it is obvious from these studies that the apparent different results in the literature are likely due to the use of different mouse models and most importantly the time frame of the induced neutropenia.

Aside from depletion studies, *C. neoformans* has also been reported in modulating the extracellular killing ability of neutrophils (217, 218). The work in these two studies implicates sphingomyelin synthase as a key enzyme in regulating the killing activity of neutrophils through the regulation of sphingomyelin production. Interestingly, the 2011 study found that neither the presence nor the size of the capsule did not influence the extracellular killing activity of these neutrophils although the GXM capsule is associated with immune modulation in many other cell types such as macrophages, monocytes, DCs, and T cells (217). The authors did find that melanized yeast cells completely abrogated the extracellular killing ability of these cells, and that live but not heat killed cells were necessary for this loss of extracellular killing activity. A key limitation in this study comes from using differentiated peripheral blood cell line, HL-60 cells, for the extracellular killing activity assays. These cells are human derived and possess increased killing ability over murine neutrophils but still derived from a propagated cell line.

##### Recruited dendritic cells

Recruited inflammatory DCs are monocyte-derived and termed monocyte-derived DCs (moDCs) (110, 116, 219). Overall, moDCs differ from resident DCs in phagocytosis and killing, cytokine release, and migration from the periphery to the nearest draining lymph nodes for activation of adaptive cell-mediated immunity (47, 198, 220–225). Upon arrival in the lung, moDCs downregulate the monocytic marker Ly6C and possess overlapping function with cDCs. The general consensus in the DC literature is less of a phenotypic classification compared to a functional classification, thus recruited DCs are believed to be an addition to the resident cDC defense arsenal rather than a functionally distinct defense (226), although moDCs have been described to be similar to cDC1 cells with a dependence on the transcription factors Irf8 and Batf3 for development, respond to IFN-γ, and express IL-2 and IL-12 (219).

In terms of anti-cryptococcal immunity, DCs have a wide repertoire of pattern recognition receptors (PRRs) that aid in recognition of fungal danger signals like capsular and cell wall components (via TLR2, TLR4, Dectin-1, and DC-SIGN), and fungal DNA upon yeast lysis (via TLR9). DCs respond with IL-2 and IL-23 production in the lung during inflammatory responses (47, 198, 219, 225, 227), thus aiding in the type 1 and 17 immune responses in the lung, respectively.

In the recent literature, the Wormley lab presented compelling evidence for the role of *in vivo* memory of DCs to *C. neoformans* (222). Although innate immunity has been widely believed to be devoid of immunological memory, the authors showed that DCs isolated from mice immunized with an IFN-γ producing H99 mutant strain (228) responded with a greatly increased type 1-based immune signature compared to naïve control mice 70 days after initial challenge. These DCs, when cultured *ex vivo*, responded with a significantly enhanced cytokine recall response of IFN-γ, IL-4, and IL-2 in response to *C. neoformans* components but not to *C. albicans, Staphylococcus aureus*, or LPS. These results not only uncover aspects of *C. neoformans* vaccination biology, but also greatly add to the growing body of literature that DCs are essential components to host defense against *C. neoformans* with a vast repertoire of anti-fungal capabilities.

##### Monocytes

Monocytes are mononuclear phagocytes that originate in the bone marrow and circulate through the blood with a variety of PRRs to enable detection of pathogens. Monocytes exist as both CCR2^+^ Ly6C^hi^ in mice (CD14+ CD16- in humans) and CCR2^lo^ Ly6C^lo^ in mice (CD14^lo^ and CD16- in humans). Ly6C^hi^ are inflammatory monocytes that extravasate into infected tissues, while Ly6C^lo^ are tissue patrolling monocytes that deal with tissue repair and homeostasis (229, 230). Monocytes respond very quickly in response to infection that succeeds neutrophils and are essentially precursors to macrophages and DCs (moDCs). Circulating monocytes have been characterized and functionally described as either mature or immature, which is highly dependent on the cytokine profile of the tissue they are infiltrating (231–236). In the presence of inflammation, mature monocytes express high levels of MHC-II and costimulatory markers such as CD80, CD86, and CD40, whereas immature monocytes express lower levels of these markers (169). However, the role of monocytes in response to *C. neoformans* infection has been debated, since the presence of monocytes can be detrimental or beneficial to the host depending on the infection model (50, 58, 237–239).

Both mature and immature monocytes can respond early on to *C. neoformans* infection. Both cells can phagocytose the fungus, however immature monocytes have decreased killing ability and harbor them back into the bloodstream across the blood brain barrier via the Trojan Horse model (240). For example, Charlier et al. intravenously infected mice with bone marrow-derived monocytes (BMDM) loaded with *C. neoformans* to demonstrate the existence of the Trojan horse model of crossing the blood brain barrier (BBB) (58). The authors found that BBB crossing was observed as early as 6 h post infection and a significant increase in the fungal burden in the spleen and kidney was observed with the yeast loaded BMDMs compared to unloaded BMDMs. These results suggest a role for monocyte trafficking of *C. neoformans* to the CNS via the Trojan horse model of infection. In addition to this, Heung and Hohl showed that CCR2+ Ly6C^hi^ inflammatory monocytes respond early on in the lung to *C. neoformans* strain H99 and aid in fungal trafficking of the yeast from the lungs to the lung draining lymph node (237). Upon depletion of these cells using CCR2-DTR mice, a significant decrease in lung fungal burden and improved host survival were observed. This was in addition to lower numbers of ILC2s, Th2 lymphocytes, and M2 macrophages observed in the lungs of CCR2-DTR mice. These data suggest that CCR2+ monocytes may skew the lung immunity toward a non-protective type 2 immune response to the highly virulent H99 strain.

Conversely, three groups have reported a role for CCR2+ cells during C. neoformans infection. First, Traynor et al. infected both CCR2 KO and WT mice with *C. neoformans* strain 52D (less virulent serotype D) and reported that CCR2 KO mice had prolonged lung fungal burden (239). In addition, CCR2 KO mice had increased eosinophilia, increased leukocyte production of IL-4 and IL-5, and lack of a delayed-type hypersensitivity response, which all signify a type 2 immune response. Second, Osterholzer et al. showed that CCR2-deficient mice infected with *C. neoformans* strain 52D exhibited prolonged infection and a type 2 immune response compared to WT mice (238). These effects were attributed to impaired DC recruitment and protective T cell polarization. Third, Masso-Silva et al. compared the immune response between an infection with the WT H99 strain and a hypovirulent mutant, *C. neoformans* Δ*fbp1* (223). From flow cytometry and lung burden analyses, the authors concluded that CCR2+ monocytes differentiate into moDCs in response to the Δ*fbp1* mutant, and that CCR2 depleted mice were not protected due to an impaired T cell response in the lungs. Together, these studies suggest that CCR2-mediated recruitment of monocytes aid in fungal clearance of the less virulent *C. neoformans* 52D or the hypovirulent strain *C. neoformans* Δ*fbp1*.

The collective conclusion from the above results in the mouse model point to monocytes being detrimental in response to the highly virulent serotype A *C. neoformans* H99, but protective in response to the weaker 52D strain. The GXM capsule of *C. neoformans* has been described as immunosuppressive (43) and has the ability to modulate macrophage polarization toward an M2 state (42, 44, 128, 241, 242). In addition to this, the five serotypes of *Cryptococcus* GXM capsule are differentially recognized by host innate immune cells, which suggests a possible variation in response to different serotypes. It is possible that an unknown GXM PAMP from serotype D strains drives monocytes into a protective maturation state, while serotype A strains either directly deter monocytes into an immature state or lack the necessary ligand needed to drive monocyte maturation.

##### Eosinophils

Eosinophils are another type of granulocyte (in addition to neutrophils) that may be recruited to the lungs during cryptococcal infection. Eosinophils are generally associated with allergic inflammation and are common effector cells against parasitic infections (243, 244), but these cells have been described as harmful in the host response to *C. neoformans* infection (135, 196, 239, 245, 246). Indeed, these studies (as well as others) have used eosinophil accumulation in the lungs as a marker of non-protective host immunity since these cells respond to increased levels of type 2 cytokines such as IL-5 and eotaxin.

In a recent study, Wiesner et al. utilized several approaches in surmising the lung lymphocyte-mediated recruitment of different granulocyte populations to the lung upon infection with the virulent *C. neoformans* strain KN99α in the C57BL/6 mice model (214). The authors used transgenic mouse models (STAT6 KO to ablate a Th2 response) and antibody depletions (anti-CD4 or anti-IL-5 to ameliorate these cells or cytokines, respectively) to determine the order of lymphocyte-mediated recruitment of granulocytes to the lungs went in order from Th2, Th17, Tc17, γδ T cells, and ILC2. In other words, Th2 CD4+ T cells from WT animals will preferentially recruit eosinophils via IL-5 in response to the virulent KN99α strain. When these cells were depleted, type 17 immune cells in order from Th17, Tc17, and γδ T cells recruited neutrophils to the lungs via IL-17A production. Not only did this study shed light upon a differential regulation of granulocyte recruitment in response to *C. neoformans* infection, but it also demonstrated a previously unstudied “pecking order” for lung cell recruitment in these mice that are notoriously susceptible to cryptococcosis. An interesting follow up to this study would involve using the less virulent serotype D strain in C57 mice as well as using both serotypes A and D in a more resistant mouse model such as CBA/J mice. This is an important concept because eosinophilia has been reported in human cryptococcosis cases (247) and the underlying cause of susceptibility of C57 mice to *C. neoformans* compared to other inbred strains such as CBA/J (39, 248, 249), which needs to be at the forefront of interpreting data across different strains of mice.

Eosinophils in rats exhibit different immunophenotypes than what is observed in mice (87, 250). In rats, eosinophils are part of the inflammatory response to *C. neoformans*. Garro et al. took an *in vitro* approach to studying the outcome of rat peritoneal eosinophils with opsonized *C. neoformans* (250). The authors found that eosinophils phagocytosed the yeast cells, upregulated MHC-I, MHC-II, and costimulatory molecules, and exhibited increased production of IL-12, TNFα, and IFN-γ. Additionally, these yeast-loaded eosinophils were able to induce CD4 and CD8 T cell proliferation and type 1 cytokine responses when cocultured together. Together these data suggest that eosinophils from rats possess anti-cryptococcal capabilities, act as antigen presenting cells, and promote a protective type 1 response.

#### Late Stage Adaptive Cell-Mediated Immunity to *C. neoformans*

Adaptive immune priming and recruitment to the lung is essential in host defense against *C. neoformans*. While B cells and antibody humoral responses show some beneficial aspects, T cell mediated immunity via CD4 and CD8 T cells provides optimal control and killing of the yeast cells. These cells express the canonical TCR with α and β subunits and require presentation of antigens on MHC molecules by APCs to become activated and recruited.

##### T Lymphocytes

T cells can be divided into CD4^+^ and CD8^+^ subsets, each possessing protective capabilities in the absence of the other during infection with *C. neoformans* (251–255). T cell-mediated immunity represents the most widely accepted potent host defense against *C. neoformans* infections, as well as the deepest downfall when a host is lymphopenic such as in HIV/AIDS patients. Indeed, T cells have been clinically implicated as major factor in the development of cryptococcal meningitis (115, 246, 256). These cells can aid in protection by either being directly cytotoxic or by secreting cytokines that aid in increased phagocyte uptake and anti-cryptococcal killing (252, 254, 257–260). Unlike the fluid polarization states of macrophages, once T cells polarize, they have more of a defined immune signature. Although differentiated T cell plasticity exists, type 1, 2, or 17 T cells (either CD4+ or CD8+) truly represent the subsets that pertain to anti-cryptococcal immunity and protection. These sub-sets are the most reported in the cryptococcal literature (expanded upon further down in this section), but a comprehensive review of this late-stage plasticity can be found in the following reviews (261–263).

The CD4^+^ T lymphocyte subsets Th1 and Th17 have been shown to be protective in response to infection with *C. neoformans*, with Th1 be more widely studied in the literature. Th1 T cells mature from naïve T cells in the presence of IFN-γ and are known to potently secrete IFN-γ and IL-2 as the major effector cytokines in response to IL-12 and IL-1β from lung macrophages to maintain the type 1 inflammatory response (258, 260, 264, 265). IFN-γ from Th1 T cells potently induces DC maturation for increased antigen presentation, classically activated M1 macrophages with increased reactive oxygen and nitrogen species production, and IgG2a/IgG3 B cell class switch recombination.

Th17 T cells are also necessary for anti-fungal defense and are defined by secretion of IL-17, which has a vast repertoire of inflammatory and tissue-specific responses. These cells mature from naïve T cells in the presence of TGF-β, IL-1, IL-23, and IL-6. IL-17 secretion from Th17 cells increases hematopoiesis and myelopoiesis, induce chemoattractants such as IL-8 and MCP-1 for neutrophil and monocyte recruitment, stimulate IgG2a/IgG3 B cell class switch recombination, and lead to downstream increases in prostaglandin E2, IL-6, nitric oxide, and IFN-γ production (120, 204, 224, 258, 266, 267). Th17 T cells also produce IL-22 in mucosal barrier tissues such as the gut and lung (268–270). Although Th22 T cells are a distinct subset, IL-22 producing Th17 cells are commonly observed in the mucosal barrier tissues. IL-22 increases the mucosal barrier integrity of these tissues, which in the case of pulmonary cryptococcosis has been suggested to prevent dissemination although this phenomenon has not been directly tested *in vivo* for *C. neoformans*.

Th2 T cells have been shown to be harmful during active cryptococcal infection. These cells mature from naïve T cells in the presence of IL-4 from alternatively activated macrophages and IL-33 lung epithelial and ILC2 cells (135, 243). These cells are seen in response to hosts that cannot control the early stages of infection when macrophages and DCs are polarized toward a type 2 state. These cells are associated with anti-parasitic defense via the secretion of IL-4, IL-5, and IL-13 and promote the recruitment of eosinophils. Altogether these cells lead to an anti-inflammatory response, M2 macrophage polarization, and persistence of fungal growth.

CD8+ T cells have also been described in antifungal defense but less so during infection with *C. neoformans* (251, 253, 267, 271–273). Analogously to the Th subsets of CD4+ T cells, the Tc subsets of CD8+ T cells have been described elsewhere (243, 251, 267) and follow the same immunological roles with Tc1, Tc17, and Tc2 mirroring Th1, Th17, and Th2, respectively. These Tc cell subsets have been reported have better direct killing ability of infected cells than the Th counterparts, however. Overall, the long-standing belief that CD4+ T cells are the sole driver of anti-cryptococcal immunity can be disregarded in our opinion since both CD8+ and CD4+ T cells possess potent anti-fungal capabilities during *C. neoformans* infection.

### Summary of Immune Response Kinetics During Primary Infection

It is obvious that there is a well-orchestrated host response in the recognition, cytokine secretion, and temporal cell recruitment to immunological control of *C. neoformans*. When the fungal particles are inhaled into the lungs, epithelial and tissue resident cells recognize and take up the fungal particles (Figure 1). In response to this uptake, early cytokines such as IL-8 and IL-1 from the epithelial cells, TNFα and IFN-γ from macrophages, and IL-2, IL-12, and IL-23 from DCs induce an inflammatory state that promotes M1 macrophage polarization and chemokine secretion, such as GM-CSF and MCP-1. The collaborative effect of these early signals induces myelopoiesis and recruitment of neutrophils, monocytes, and DCs to the lung. In the presence of macrophage-derived IFN-γ and TNFα, DCs mature, phagocytose fungal particles, upregulate costimulatory molecules and migration markers, and migrate to the mLN for antigen presentation to naïve T cells. During this time, γδ T cells and ILCs may also play a role in the coordination the immune response, although their exact role is still unclear. As the innate immunity controls fungal proliferation, the adaptive immune system is recruited to the lung where type 1/17 T cells become potent sources of IFN-γ, IL-2, and IL-17 that activate the killing and/or containment of the yeast cells. Epithelial barrier integrity and antimicrobial peptide production are stimulated by IL-22 to deter extrapulmonary dissemination, phagocytic killing abilities are increased, and anti-fungal neutrophils are recruited and activated, leading to total elimination of fungal cells or/and containment of the yeast in a granuloma. Altogether, these cells work together synergistically for optimal host control of the infection.

### Reactivation Model of a Latent Fungal Infection: Breakdown of Granuloma Containment

There are several noteworthy differences from the primary infection immune response previously described in mice and the response to reactivated *C. neoformans*. First, in the reactivation model, the fungal cells are already present in the lung tissue. Second, the host has already experienced a pulmonary cryptococcal infection, contained it, and has developed adaptive immunity against the yeast. Third, the host was able to control the initial infection during an immunocompetent state. Yet, if the host is experiencing reactivation of latent yeast cells, then some immunosuppressive event has led to this reactivation nullifying one or more host cell types that normally control the fungal replication.

With this said, the reactivation model is still one of the most understudied topics in regard to cryptococcosis mostly due to the lack of tools in model organisms. The mouse is by far the most popular animal model organism to study the primary infection model of cryptococcosis, yet the mouse not being able to form lung granulomas for containment of the fungus that recapitulate human granulomas results in the reactivation model being understudied (274). Mice have been previously mentioned to form a granulomatous response to moderately virulent *C. neoformans* strain 52D that forms a persistent infection. Although certain transgenic models have been shown to form granulomas, an immunocompetent mouse does not.

However, studies in our lab found that when mice are infected with a *C. neoformans* mutant strain in which the glucosylceramide synthase gene has been deleted (Δ*gcs1*), they formed lung granulomas that closely resembled human lung granulomas (111). These granulomas are depicted by a necrotic center, a characteristic ring of foamy macrophages and multi-nucleated giant cells loaded with fungi or fungal debris (Figure 2). The macrophage ring is surrounded by an infiltration of lymphocytes, fibroblasts, and fibrotic tissue with collagen deposition. The resemblance of this mouse granuloma with the human lung granuloma (275–277) is simply striking.

In follow up studies with this mutant, the formation of these lung granulomas to contain *C. neoformans* Δ*gcs1* was found to be dependent on the host enzyme sphingosine kinase 1 (SK1) in the lungs (24, 274). Elevated levels of S1P, MCP-1, and TNFα in the bronchoalveolar lavage fluid were found to be significantly associated with granuloma containment. Interestingly, during the infection with Δ*gcs1*, lung macrophages are a significant source of S1P, MCP-1, and TNFα and a highly regarded cell type in granuloma homeostasis. S1P is a widely studied immunological signaling molecule that directs leukocyte migration and function (278, 279), MCP-1/CCL2 is a pro-inflammatory cytokine that aids in recruitment of CCR2^+^ monocytes, memory T cells, and DCs, and TNFα is a pro-inflammatory cytokine that activates phagocytes and aids in DC maturation for induction of T cell-mediated immunity. Taken together, macrophages have been shown here to exert an early signaling cascade that is dependent on SK1 and drives the recruitment of innate and adaptive immunity to begin granuloma containment of *C. neoformans* Δ*gcs1*.

Sphingosine phosphate receptors became an important function in granuloma reactivation since this family of five receptors are heterogeneously distributed on immune cells and respond to S1P gradients [for a detailed review of this topic please see (280, 281)].

Certain medications can cause immunosuppression and thus the prolonged administration of these medications is a predisposing factor for developing cryptococcosis. However, whether cryptococcosis develops as a result of the primary infection or reactivation is still controversial. One medication that causes a dramatic T cell depletion is FTY720 (Gilenya) used to treat multiple sclerosis (282).

Upon administration, FTY720 is rapidly phosphorylated into FTY720-P, becoming an analog of S1P and now able to bind S1P receptors 1, 3, 4, and 5. Other FTY720 derivatives, such as BAF312, is able to directly bind its receptors (1, 4, and 5) without being phosphorylated. Bryan et al. found that treatment with FTY720 but not BAF312 causes cryptococcosis in mice, and the infection is a result of a reactivation from the granuloma rather than a dissemination from the primary infection (283).

Very interestingly, FTY720 treated mice exhibited altered granuloma structure and impaired macrophage killing activity compared to BAF312 treated mice, despite the observation that both drugs caused similar lymphopenia. In addition, compared to BAF312, FTY720 not only disorganized the ring of macrophages at the granuloma site but these macrophages were mostly M2 polarized. These phenotypes are linked to the specific interaction of FTY720 to S1Pr3 (not targeted by BAF312), as macrophages lacking S1Pr3 also displayed poor phagocytosis and killing activity.

The observation that lymphopenia was present during both treatments clearly suggests that this immune condition is necessary but not sufficient to reactivate latent *C. neoformans* during FTY720 treatment (283).

It is important to mention that a limitation of this study is the use of *C. neoformans* Δ*gcs1* mutant. *C. neoformans* Δ*gcs1* mutant lacks the sphingolipid glucosylceramide. As a result, the mutant goes in cell cycle arrest in neutral/alkaline pH; it does not die when cells are exposed to these environments, rather it just cannot replicate (111). On the other hand, fungal glucosylceramides, including the species produced by *C. neoformans*, are highly immunogenic and mice develop antibodies against this fungal sphingolipid (284). It is possible that this antibody production in mice is counterproductive for the development of the granuloma, although antibody production also occurs in humans (285), whom are capable to develop lung granulomas. Due to its immunogenicity, fungal glucosylceramide may alter the initial lung innate immunity leading to the granuloma formation, in addition to have a notable effect on restricting fungal replication.

Nonetheless, the work using this cryptococcal mutant (30, 274) have laid the foundation for important animal studies in the reactivation of *C. neoformans*, such as the recent work by Bryan et al. (283) on the reactivation of the cryptococcal disease under certain drug immunodeficiency. The fact that macrophage function at the granuloma site must also be altered in addition to lymphopenia, it may explain why only a small fraction of subjects with AIDS develop cryptococcosis.

## Conclusions and Future Perspectives

With the ever-increasing population of immunocompromised individuals, improvements in treatment and vaccinations are imperative for opportunistic infections, such as cryptococcosis. These improvements begin with structuring proper animal model systems to study the infection kinetics on the basic research side before being implemented into the clinical side. Work using the primary infection model purports invaluable information for protective anti-cryptococcal immunity and more research on certain host cell types, such as epithelial cells, γδ T cells, and ILCs, is warranted. This is especially important for investigators that look into protective immunity elicited by vaccination strains, since a thorough understanding of these models is at the forefront of transitioning from basic research into human clinical trials.

Also, the reactivation model of cryptococcosis has been widely understudied. The *C. neoformans* Δ*gcs1* mutant offers an invaluable tool that opens up new possibilities, but caution should be taken because the effect of glucosylceramide on the host immunity is not fully understood. Nonetheless, the observation that a drug (FTY720) used to treat reoccurring multiple sclerosis resulted in *C. neoformans* reactivation, has paved the way for a better understanding of the lung immunity required for both the formation and the containment of the lung granuloma.

## Author Contributions

TN and MD contributed to the initial writing. TN, AB, and MD contributed to the editing and finalization of the manuscript. All authors contributed to the article and approved the submitted version.

## Conflict of Interest

MD is a Co-Founder and Chief Scientific Officer (CSO) of MicroRid Technologies Inc. AB is an employee of inGenious Targeting Laboratory Inc. The remaining author declares that the research was conducted in the absence of any commercial or financial relationships that could be construed as a potential conflict of interest.
